# The Association between Carbohydrate-Rich Foods and Risk of Cardiovascular Disease Is Not Modified by Genetic Susceptibility to Dyslipidemia as Determined by 80 Validated Variants

**DOI:** 10.1371/journal.pone.0126104

**Published:** 2015-04-21

**Authors:** Emily Sonestedt, Sophie Hellstrand, Christina-Alexandra Schulz, Peter Wallström, Isabel Drake, Ulrika Ericson, Bo Gullberg, Bo Hedblad, Marju Orho-Melander

**Affiliations:** 1 Diabetes and Cardiovascular Disease—Genetic Epidemiology, Department of Clinical Sciences Malmö, Lund University, Malmö, Sweden; 2 Nutritional Epidemiology, Department of Clinical Sciences Malmö, Lund University, Malmö, Sweden; 3 Cardiovascular Disease Epidemiology, Department of Clinical Sciences Malmö, Lund University, Malmö, Sweden; University of East Anglia, UNITED KINGDOM

## Abstract

**Background:**

It is still unclear whether carbohydrate consumption is associated with cardiovascular disease (CVD) risk. Genetic susceptibility might modify the associations between dietary intakes and disease risk.

**Objectives:**

The aim was to examine the association between the consumption of carbohydrate-rich foods (vegetables, fruits and berries, juice, potatoes, whole grains, refined grains, cookies and cakes, sugar and sweets, and sugar-sweetened beverages) and the risk of incident ischemic CVD (iCVD; coronary events and ischemic stroke), and whether these associations differ depending on genetic susceptibility to dyslipidemia.

**Methods:**

Among 26,445 individuals (44–74 years; 62% females) from the Malmö Diet and Cancer Study cohort, 2,921 experienced an iCVD event during a mean follow-up time of 14 years. At baseline, dietary data were collected using a modified diet history method, and clinical risk factors were measured in 4,535 subjects. We combined 80 validated genetic variants associated with triglycerides and HDL-C or LDL-C, into genetic risk scores and examined the interactions between dietary intakes and genetic risk scores on the incidence of iCVD.

**Results:**

Subjects in the highest intake quintile for whole grains had a 13% (95% CI: 3–23%; p-trend: 0.002) lower risk for iCVD compared to the lowest quintile. A higher consumption of foods rich in added sugar (sugar and sweets, and sugar-sweetened beverages) had a significant cross-sectional association with higher triglyceride concentrations and lower HDL-C concentrations. A stronger positive association between a high consumption of sugar and sweets on iCVD risk was observed among those with low genetic risk score for triglycerides (p-interaction=0.05).

**Conclusion:**

In this prospective cohort study that examined food sources of carbohydrates, individuals with a high consumption of whole grains had a decreased risk of iCVD. No convincing evidence of an interaction between genetic susceptibility for dyslipidemia, measured as genetic risk scores of dyslipidemia-associated variants, and the consumption of carbohydrate-rich foods on iCVD risk was observed.

## Introduction

It is still unclear whether carbohydrate consumption is associated with cardiovascular disease (CVD) risk. Evidence suggests that carbohydrate quality is more important than carbohydrate quantity, and that in addition to macronutrients it is important to examine foods rich in carbohydrates. For example, whole grains are related to a decreased risk of CVD [1, 2], whereas sugar-sweetened beverages are associated with an increased risk of CVD [3, 4]. Previously, in the Malmö Diet and Cancer study (MDCS), we have shown that high fiber consumption associates with protection against CVD [5], and men adhering to the recommended fruit, vegetable and sugar consumption guidelines had a decreased risk for CVD [6]. One of the proposed mechanisms by which dietary intakes can influence CVD risk is through their impact on blood lipids and lipoproteins. In the MDCS, sugar consumption was positively related to the atherogenic lipid profile which was composed of high levels of triglycerides and small LDL and low levels of HDL-C [7]. It is possible that genetic susceptibility to dyslipidemia and CVD risk may influence the association between dietary intake and risk of CVD. In the recent years, genome-wide screening for common variants that associate with blood lipids and lipoproteins has made great progress. In 2010, 95 validated genetic loci were identified, which together accounted for 10–12% of the total variance and 25–30% of the genetic variance, in blood lipid and lipoprotein levels [8]. Recently, an additional 62 genetic loci were identified accounting for another 2–3% of the variance [9]. One study demonstrated an interaction on the lipid-lowering effect between a weight-loss intervention and genetic risk score for total dyslipidemia composed of 32 lipid-associated genetic variants [10]. However, because carbohydrates might show associations with specific lipid traits, it is important to examine risk scores that reflect the genetic susceptibility for adverse levels of triglycerides, HDL-C and LDL-C separately. Therefore, the primary objectives of this study were to 1) examine the association between consumption of different carbohydrate-rich foods (vegetables, fruits and berries, juice, potatoes, whole grains, refined grains, cookies and cakes, sugar and sweets, and sugar-sweetened beverages) and risk of incident ischemic CVD (iCVD) in the MDCS cohort and 2) examine whether genetic susceptibility to dyslipidemia, measured as the genetic risk scores of 80 validated variants, modify such associations. As a secondary objective we examined the cross-sectional associations between the consumption of carbohydrate-rich foods and clinical risk factors of iCVD at baseline (blood pressure, triglycerides, HDL-C, LDL-C and insulin resistance) and whether the associations with blood lipids and lipoproteins were modified by genetic susceptibility to dyslipidemia.

## Subjects and Methods

### Subjects and data collection

The Malmö Diet and Cancer Study (MDCS) is a prospective population-based cohort with baseline examinations conducted between 1991 and 1996. All men born between 1923 and 1945 and women born between 1923 and 1950 that live in Malmö were invited via personal letters and advertisements in the local newspaper and public places to participate in the study. Limited Swedish language skills and mental incapacity were the only exclusion criteria. The participation rate was approximately 40% [11]. The ethical committee at Lund University approved the study (LU 51–90), and the participants gave their written informed consent. Dietary intakes were collected using a modified diet history method. Information regarding different lifestyle and demographic factors (including education, smoking, leisure-time physical activity, and drug use) was collected using a self-administered questionnaire. Nurses measured blood pressure and body composition and collected blood samples. In total, 28,098 individuals completed the lifestyle questionnaire and diet assessment and had their anthropometrics measured. After excluding those with a history of myocardial infarction, stroke, or diabetes, 26,445 individuals remained and constituted the study sample for this study. A 50% random sample (n = 6,103) of those that participated between 1992 and 1994 were invited for an additional visit (on average 0.7 years after the previous visit) for collecting fasting blood samples. Blood concentrations of fasting triglycerides, HDL-C, glucose and insulin were measured with standard procedures, and LDL-C concentration was calculated using the Friedewald formula: LDL = total cholesterol—HDL-C—(triglycerides/2.2). Homeostasis model assessment (HOMA-IR) was used as a measure of insulin resistance and was calculated using the following formula: (fasting insulin x fasting blood glucose)/22.5.

### Dietary data

Usual dietary intakes were estimated using a modified diet history method specifically developed for MDCS [12]. Information on cooked lunches and dinners and cold beverages was collected over 7 consecutive days, and other foods regularly consumed during the past year were collected through a 168-item dietary questionnaire, with frequencies of food intake and usual portion sizes assessed using photographs. During a 1-hour interview, the participants were asked questions about their food choices, food preparation practices and portion sizes of the foods they consumed during the 7 days studied. The routines for coding dietary data were slightly altered in September 1994 in order to shorten the interview time. This change did not reveal any major influence on the ranking of individuals [13]. A variable was constructed (“diet method version”) to distinguish between data collection before and after the change in diet assessment method. Energy and nutrient intakes were computed from the reported food intakes using the MDCS Food and Nutrient Database, mainly originating from PC Kost2-93 of the Swedish National Food Administration in Uppsala.

The nutrients examined in this study were: total carbohydrates, sucrose and dietary fiber. The foods examined were selected for being significant sources of dietary carbohydrates: vegetables (g/day), fruits and berries (g/day), juice (g/day), potatoes (g/day), whole grains (portions/day of fiber-rich bread, biscuits and cereals), refined grains (portions/day of low-fiber bread, biscuits, cereals, rice and pasta), cookies and cakes (g/day), sugar and sweets (g/day), and sugar-sweetened beverages (g/day). Milk, which is also a significant source of carbohydrates, has already been examined in relation to iCVD risk within MDCS and therefore was not included in this study [14]. The nutrient and food intakes were adjusted for total energy intake using the residual method (ln-transformed food intake as the dependent variable and ln-transformed total energy intake as the independent variable). The residuals were divided into quintiles for most of the nutrients and foods. For the foods with >25% zero-consumers (i.e., juice and sugar-sweetened beverages), we categorized zero-consumers into a separate group and divided the rest of the individuals into tertiles according to their energy-adjusted intakes. The validity and reproducibility of the dietary method have been examined previously [15–17]. Energy-adjusted Pearson’s validity correlation coefficients (compared to 18 days of weighed food records) were for carbohydrates (0.66 and 0.70 for men and women, respectively), fiber (0.74/0.69), sugar (0.60/0.74), vegetables (0.65/0.53), fruits (0.60/0.77), potatoes (0.69/0.51), cereals (0.74/0.73), breads 0.50/0.58), rice and pasta (0.35/0.24) [16, 17].

### Cardiovascular endpoints

Incident CVD events were identified by linkage to the Swedish Hospital Discharge Registry and Cause-of-death Registry [18]. Stroke events were also identified from the local stroke registry in Malmö (STROMA) [19]. Coronary heart disease was defined as ICD-9 codes 410–414 (fatal or non-fatal myocardial infarction or death due to ischemic heart disease). Ischemic stroke was defined as ICD-9 code 434 and diagnosed when computed tomography or autopsy could verify the presence of an infarction and/or exclude hemorrhage and non-vascular disease. Vital status and emigration was extracted from the National Tax Board. Until the end of follow-up on June 30, 2009, 2,921 of the study participants were diagnosed with an iCVD event. The participants contributed to time of follow-up until iCVD diagnosis, emigration, death or end of follow-up. Cases with intra-cerebral (ICD-9 code 431), subarachnoid hemorrhages (ICD-9 code 430) or non-specific stroke (ICD-9 code 436) were not included in the analyses with iCVD, because the risk factors are different compared to coronary heart disease and ischemic stroke; however, they contributed to person-years of follow-up until their diagnosis.

### Genotyping and construction of genetic risk scores

Genotyping was performed at the Clinical Research Centre, Malmö, Sweden, by using the Sequenom MassARRAY (Sequenom, San Diego, CA, USA) or Taqman allelic discrimination on an ABI 7900 (Applied Biosystems, Foster City, CA, USA). We estimated susceptibility for dyslipidemia by combining the validated single-nucleotide polymorphisms (SNP) reported in the genome-wide association meta-analysis by Teslovich et al. [8]. We genotyped all SNPs (n = 91) that had reached the genome-wide significant level for either triglycerides, HDL-C or LDL-C, except *LPA* rs1084651, *JMJD1C* rs10761731 and *NPC1L1* rs217386 because of difficulties in genotyping or no proxies were available. SNPs were then excluded in the current study if the success rate was less than 90% (*COBLL1* rs10195252, *KLF14* rs4731702, *PLEC1* rs11136341, *ABCA8* rs4148008) or if the Hardy-Weinberg equilibrium p-value was less than 0.00057 (0.05/87) (*ANGPTL3* rs2131925, *TYW1B* rs13238203, *SCARB1* rs838880, *OSBPL7* rs7206971, *LILRA3* rs386000, *PLTP* rs6065906, *MOSC1* rs2642442). Weighted genetic risk scores were constructed using PLINK (version 1.07) for triglycerides (26 SNPs), HDL-C (41 SNPs) and LDL-C (32 SNPs) by multiplying the effect size (β-coefficients) found in the meta-analysis [8] with the number of risk alleles and then summing the products. In these analyses, those 24,799 individuals who had more than 60% of their SNPs successfully genotyped were included.

### Other variables

Body mass index (BMI; kg/m^2^) was calculated from direct measurements of weight and height. Subjects, wearing light clothing and no shoes, were weighed using a balance-beam scale for weight and a fixed stadiometer for height (cm). Smoking habits were categorized into smokers, ex-smokers and never smokers. Alcohol consumption was categorized into six categories. Individuals indicating no consumption during the past year and no consumption in the 7-day food diary were categorized as zero-consumers. The other individuals were divided into gender-specific quintiles based on their consumption in the food diary. Education level was divided into elementary, primary and secondary, upper secondary, further education without a degree, and university degree. Leisure-time physical activity was obtained from the questionnaire where the individuals had to estimate minutes per week they spent on 17 different activities. A score was constructed taking into account the intensity and duration of the activity; the score was divided into quintiles. Non-adequate reporters of energy were determined by comparing their reported energy intake with their total energy expenditure (estimated from their calculated basal metabolic rate and self-reports of leisure-time physical activity, work activity, household work, and sleep hours). Individuals with reported energy intake above or below the 95% confidence interval for total energy expenditure were categorized as “misreporters”. Individuals answering yes to the questionnaire item “Have you substantially changed your dietary habits in the past?” were classified as “dietary changers”.

### Statistical methods

SPSS (version 20, IBM Corporation, Armonk, NY) was used for all statistical analyses. Baseline characteristics according to incident iCVD status were examined using general linear model adjusted for age and sex. The mean age, BMI and dietary intakes of the lowest and highest intake groups in the food categories (quintiles or zero-consumers/tertiles) were reported separately for men and women. We also examined the linear relationship between the intake of food group categories and possible mediating risk factors between dietary intake and iCVD risk (i.e., blood pressure, fasting blood lipids, lipoproteins, and HOMA-IR) among the 4535 individuals with complete data on all risk factors and the variables that were adjusted for (i.e., age, sex, season of data collection [spring, summer, winter, autumn], total energy intake, BMI, smoking habits, alcohol consumption, leisure-time physical activity and education). Blood pressure analyses were also adjusted for the use of anti-hypertensive drugs, and blood lipid and lipoprotein analyses were also adjusted for the use of lipid-lowering drugs. We used ln-transformed risk factor variables to test for trend across food group categories. In sensitivity analyses, we excluded individuals categorized as misreporters of energy intake (18% of the study sample, see above).

Cox proportional hazard regression analysis was used to calculate hazard ratios for iCVD with the lowest food category (lowest quintile) as the reference, and with person-years of follow-up as the underlying time-variable. The proportional hazards assumption was checked graphically using log minus-log survival plot and tested using Shoenfeld residuals. These analyses were adjusted for age, sex, season of data collection, diet method version, total energy intake, and several potential confounders (i.e., BMI, smoking habits, alcohol consumption, leisure-time physical activity, and education). These covariates were selected a priori for being known risk factors for CVD and for being associated with dietary habits, without being considered intermediates between dietary habits and disease. If values for any of the categorical variables were missing, they were treated as a separate category (i.e., 60 individuals for smoking habits, 171 individuals for leisure-time physical activity, and 60 individuals for education). If values for the continuous variables were missing, they were excluded (i.e., 30 individuals for BMI). To examine the heterogeneity of effect in men and women we included the cross product of sex and diet exposure in the analyses. Because BMI can be regarded as a mediating factor, we also performed the analyses without BMI as a covariate in the multivariable model. In additional analyses, other dietary factors (i.e., fermented milk [g/day], coffee [g/day], meat [g/day], fish [g/day], vegetables [g/day] and whole grains [portions/day]) were included as covariates, because these dietary factors have previously been associated with iCVD risk and could confound the examined associations [14, 20]. We also included systolic blood pressure and use of anti-hypertensive drugs (yes/no) in a multivariate model. In sensitivity analyses, we excluded individuals reporting dietary changes in the past (“dietary changers”; 22% of the study sample), because these individuals were suspected to have unstable food habits [21, 22]. We also examined the associations after excluding cases that were diagnosed within two years after the baseline examination. Separate analyses were also performed with coronary heart disease or ischemic stroke as first iCVD endpoint.

We examined whether the associations between dietary intakes and iCVD risk were different depending on genetic risk scores for triglycerides, HDL-C and LDL-C. The interactions between dietary intakes and risk scores were examined by including a cross product of the continuous variables (diet categories x genetic risk score) in the multivariable model in addition to the main variables.

## Results

### Participant characteristics

The baseline characteristics of incident iCVD cases and non-cases are shown in Table 1. The food intakes and lifestyle factors in the lowest and highest food group categories are shown in S1 Table.

**Table 1:** Baseline characteristics according to cardiovascular event at follow-up on June 30, 2009.

Variables	n	Incident iCVD cases(n = 2,921)	Non iCVD cases(n = 23,524)	P^a^
Women (%)	26,445	42%	65%	
Age, y	26,445	61.8 (61.5, 62.0)^b^	57.4 (57.3, 57.5)	
BMI, kg/m^2^	26,415	26.2 (26.0, 26.3)	25.6 (25.5, 25.6)	**<0.001**
Diastolic blood pressure, mmHg	26,405	87.7 (87.3, 88.0)	85.2 (85.1, 85.4)	**<0.001**
Systolic blood pressure, mmHg	26,407	146 (145, 147)	140 (140, 140)	**<0.001**
Triglycerides, mmol/L	5,070	1.44 (1.37, 1.50)	1.33 (1.31, 1.35)	**0.003**
HDL-C, mmol/L	5,021	1.31 (1.28, 1.34)	1.40 (1.39, 1.41)	**<0.001**
LDL-C, mmol/L	4,957	4.24 (4.16, 4.33)	4.16 (4.13, 4.19)	0.09
HOMA-IR	4,624	1.80 (1.68, 1.91)	1.57 (1.53, 1.61)	**<0.001**
Energy intake, kcal	26,445	2272 (2251, 2294)	2281 (2273, 2288)	0.46
Carbohydrate, E%	26,445	45.1 (44.9, 45.4)	45.2 (45.1, 45.3)	0.75
Sucrose, E%	26,445	8.83 (8.70, 8.96)	8.60 (8.55, 8.64)	**0.001**
Fiber, g/1000 kcal	26,445	8.82 (8.73, 8.92)	9.06 (9.03, 9.09)	**<0.001**
Vegetables, g/day	26,445	175 (172, 179)	182 (180, 183)	**0.001**
Fruits and berries, g/day	26,445	189 (184, 194)	196 (194, 197)	**0.006**
Juice, g/day	26,445	61.0 (57.2, 64.8)	62.5 (61.2, 63.8)	0.47
Potatoes, g/day	26,445	123 (120, 125)	121 (120, 122)	0.14
Whole grains, portions/day	26,445	0.92 (0.89, 0.96)	0.98 (0.97, 0.99)	**0.006**
Refined grains, portions/day	26,445	2.61 (2.56, 2.67)	2.60 (2.59, 2.62)	0.76
Cookies and cakes, g/day	26,445	36.2 (35.0, 37.3)	37.4 (37.0, 37.8)	0.05
Sugar and sweets, g/day	26,445	42.1 (41.0, 43.3)	40.9 (40.5, 41.3)	0.05
Sugar-sweetened beverages, g/day	26,445	82.4 (76.9, 87.8)	76.7 (74.8, 78.6)	0.06

^a^The difference in baseline characteristics between cases and non-cases was tested with a general linear model adjusted for age and sex.

^b^All values are expressed as means (95% CI).

### The association between dietary intakes and clinical risk factors

The cross-sectional associations between food intakes and blood pressure, blood lipids and lipoproteins, and HOMA-IR were investigated among 4,535 individuals at baseline (Table 2). Total carbohydrate consumption was positively associated with triglyceride concentrations (mean [SE] = 1.33 [0.02] and 1.25 [0.02] mmol/L for highest vs. lowest quintile, p-trend = 0.003) and negatively associated with HDL-C concentration (1.35 [0.01] and 1.46 [0.01] mmol/L for highest vs. lowest quintile, p-trend = 1x10^-11^). A higher consumption of foods rich in added sugar (sugar and sweets, and sugar-sweetened beverages) was significantly associated with higher triglyceride concentrations and lower HDL-C concentrations. Specifically, mean triglyceride concentration in the highest vs. lowest quintile of sugar and sweets and sugar-sweetened beverages were 1.32/1.23 (p-trend = 0.007) and 1.37/1.25 (p-trend = 5 x 10^-6^), respectively, and mean HDL-C were 1.36/1.43 (p-trend = 3 x 10^-5^) and 1.35/1.41 (p-trend = 1 x 10^-4^), respectively. Sugar-sweetened beverage consumption was associated with higher insulin resistance (measured by HOMA-IR), while a high consumption of fiber, vegetables, and fruits and berries was associated with lower insulin resistance. Generally, the exclusion of misreporters produced stronger risk estimates. However, the results of the sensitivity analysis would not change our interpretation of the data and are therefore not shown.

**Table 2:** The associations between risk factors and energy-adjusted food intakes at baseline among 4,535 individuals in the Malmö Diet and Cancer cohort, 1992–1994.^a^

	Diastolic BP^b^		Systolic BP		Triglycerides		HDL-C		LDL-C		HOMA index	
	β^c^ (SE)	P_trend_	β^c^ (SE)	P_trend_	β^c^ (SE)	P_trend_	β^c^ (SE)	P_trend_	β^c^ (SE)	P_trend_	β^c^ (SE)	P_trend_
Carbohydrates	-0.12 (0.10)	0.24	0.30 (0.20)	0.13	0.019 (0.007)	**0.003**	-0.025 (0.004)	**1 x 10** ^**–11**^	-0.008 (0.011)	0.46	-0.033 (0.013)	**0.009**
Sucrose	0.22 (0.10)	**0.03**	0.23 (0.19)	0.23	0.026 (0.006)	**4 x 10** ^**–5**^	-0.022 (0.004)	**4 x 10** ^**–10**^	0.009 (0.011)	0.39	0.019 (0.012)	0.13
Fiber	-0.15 (0.10)	0.14	-0.13 (0.19)	0.51	-0.008 (0.006)	0.19	<0.001 (0.004)	0.94	-0.003 (0.011)	0.78	-0.056 (0.012)	**6 x 10** ^**–6**^
Vegetables	-0.06 (0.09)	0.51	-0.18 (0.18)	0.32	-0.008 (0.006)	0.20	0.009 (0.003)	**0.01**	0.011 (0.010)	0.29	-0.031 (0.012)	**0.009**
Fruits and berries	-0.13 (0.10)	0.20	-0.30 (0.19)	0.12	-0.003 (0.006)	0.61	<0.001 (0.004)	0.96	-0.005 (0.011)	0.64	-0.039 (0.013)	**0.002**
Juice	0.10 (0.11)	0.40	0.30 (0.22)	0.17	-0.002 (0.007)	0.76	-0.002 (0.004)	0.67	0.011 (0.013)	0.36	-0.022 (0.014)	0.12
Potatoes	0.12 (0.09)	0.20	0.61 (0.18)	**0.001**	0.005 (0.006)	0.43	-0.004 (0.003)	0.24	-0.003 (0.010)	0.79	0.012 (0.012)	0.32
Whole grains	0.11 (0.09)	0.23	0.04 (0.18)	0.82	-0.007 (0.006)	0.22	0.001 (0.003)	0.83	0.007 (0.010)	0.50	-0.016 (0.012)	0.17
Refined grains	-0.29 (0.09)	**0.002**	-0.14 (0.18)	0.46	-0.001 (0.006)	0.90	-0.001 (0.003)	0.69	-0.007 (0.010)	0.47	-0.016 (0.012)	0.17
Cookies and cakes	-0.14 (0.10)	0.16	-0.34 (0.19)	0.07	0.005 (0.006)	0.46	-0.01 (0.004)	**0.003**	0.028 (0.011)	**0.009**	-0.012 (0.012)	0.32
Sugar and sweets	0.14 (0.10)	0.15	0.17 (0.19)	0.38	0.017 (0.006)	**0.007**	-0.015 (0.004)	**3 x 10** ^**–5**^	-0.007 (0.011)	0.52	0.012 (0.012)	0.31
Sugar-sweetened beverages	0.18 (0.12)	0.11	0.45 (0.23)	**0.04**	0.034 (0.007)	**5 x 10** ^**–6**^	-0.016 (0.004)	**1 x 10** ^**–4**^	0.024 (0.013)	0.07	0.035 (0.015)	**0.02**

^a^The general linear model was used to test the linear relationship adjusted for age, sex, season, energy intake, BMI, smoking, alcohol consumption, leisure-time physical activity, and education. Food intakes and blood pressure analyses were also adjusted for the use of anti-hypertensive therapy; food intakes and blood lipids analyses were also adjusted for the use of lipid-lowering therapies.

^b^BP, blood pressure.

^c^β (regression coefficient) represents the mean change in biological risk markers for each increase in intake category.

### The association between dietary intakes and iCVD risk

We observed similar incidence rates of iCVD in the highest vs. lowest quintiles of total carbohydrates (8.2 vs. 8.4 cases/1000 person-years). In the basic model, sucrose consumption was positively associated with iCVD risk, while the consumption of fiber, vegetables, fruits and berries, juice, whole grains, and cookies and cakes was negatively associated with iCVD risk. When we adjusted for several lifestyle factors, only higher consumption of fiber and whole grains remained statistically significantly associated with a decreased risk of iCVD (Table 3). In the lowest quintile of whole grains (mean intake of 0.0 portions/day), there were 10.2 iCVD cases per 1000 person-years of follow-up compared to 7.8 cases per 1000 person-years of follow-up in the highest quintile (mean intake of 2.5 portions/day). The highest intake quintile of whole grains was associated with a 13% lower risk of iCVD compared to the lowest quintile. We observed no significant heterogeneity in the results between men and women for any of the food groups (all p-interaction ≥0.19). The highest whole grain intake was associated with an 8% (p-trend = 0.13) lower risk in men and a 21% (p-trend = 0.002) lower risk in women (p-interaction with sex: 0.19) for iCVD (S2 Table). Excluding BMI as a covariate or additional adjustments for several dietary factors or systolic blood pressure and anti-hypertensive drug use did not influence the risk estimates (data not shown). Excluding cases diagnosed within two years after baseline only slightly affected the results. The association with sucrose was slightly strengthened (p-trend = 0.06); however, the association was only observed among men (p-trend = 0.02) but not among women (p-trend = 0.95). Specifically, men in the highest quintile had a 20% higher iCVD risk compared to men in the lowest quintile. When we excluded individuals who reported a substantial dietary change in the past, the associations observed with intake levels of fiber and whole grains were somewhat stronger (HR for highest vs. lowest fiber quintile: 0.81; 95% CI: 0.70, 0.94; p-trend = 0.001; HR for highest vs. lowest whole grains quintile: 0.81; 95% CI: 0.71, 0.92; p-trend = 0.0001). We observed a somewhat stronger association with ischemic stroke than coronary events for dietary fiber and whole grain consumption (p-trend [fiber] = 0.02 and 0.18, and p-trend [whole grains] = 0.006 and 0.10 for ischemic stroke and coronary event, respectively) (S3 Table).

**Table 3:** Hazard ratios (95% CI) of incident cardiovascular disease by categories of carbohydrates and food groups, Malmö Diet and Cancer cohort, 1991–2009.

	Categories of intake									
	1	2		3		4		5		P_trend_
Carbohydrates										
Cases/follow-up	602/72032	546/72974		580/73008		590/73301		603/73619		
Mean intakes (E%)	37	42		45		48		53		
Basic model^a^	1.00	0.89	0.79, 1.00	0.91	0.81, 1.02	0.91	0.81, 1.02	0.92	0.82, 1.03	0.27
Extended multivariable model^b^	1.00	0.94	0.84, 1.06	0.98	0.87, 1.10	1.01	0.89, 1.14	1.00	0.88, 1.13	0.65
Excluding diet changers^c^	1.00	0.94	0.83, 1.07	0.97	0.85, 1.11	0.98	0.85, 1.12	0.96	0.83, 1.11	0.80
Sucrose										
Cases/follow-up	631/72294	528/73978		574/73457		545/73527		643/71677		
Mean intakes (E%)	4	7		8		10		14		
Basic model	1.00	0.86	0.76, 0.96	0.95	0.85, 1.06	0.90	0.80, 1.01	1.11	0.99, 1.24	**0.05**
Extended multivariable model	1.00	0.92	0.81, 1.03	1.02	0.91, 1.14	0.94	0.84, 1.06	1.08	0.96, 1.21	0.18
Excluding diet changers	1.00	0.92	0.80, 1.05	1.03	0.90, 1.17	0.93	0.81, 1.07	1.07	0.94, 1.22	0.33
Fiber										
Cases/follow-up	707/69571	603/71873		564/73191		526/74341		521/75958		
Mean intakes (g/1000 kcal)	6	8		9		10		13		
Basic model	1.00	0.83	0.74, 0.92	0.77	0.69, 0.87	0.70	0.62, 0.78	0.70	0.62, 0.79	**1 x 10** ^**–11**^
Extended multivariable model	1.00	0.91	0.81, 1.01	0.90	0.80, 1.01	0.84	0.75, 0.94	0.88	0.78, 0.99	**0.01**
Excluding diet changers	1.00	0.91	0.80, 1.02	0.88	0.78, 1.00	0.82	0.72, 0.93	0.81	0.70, 0.94	**0.001**
Vegetables										
Cases/follow-up	735/69394	649/72127		562/73192		519/74340		456/75881		
Mean intakes (g/day)	72	123		164		213		332		
Basic model	1.00	0.92	0.83, 1.03	0.85	0.76, 0.95	0.84	0.75, 0.94	0.80	0.71, 0.90	**2 x 10** ^**–5**^
Extended multivariable model	1.00	0.97	0.87, 1.08	0.93	0.83, 1.04	0.95	0.85, 1.07	0.94	0.83, 1.06	0.24
Excluding diet changers	1.00	0.98	0.87, 1.11	0.89	0.79, 1.01	0.94	0.82, 1.07	0.95	0.82, 1.09	0.25
Fruits and berries										
Cases/follow-up	656/70657	623/71997		614/72705		519/74752		509/74822		
Mean intakes (g/day)	53	120		173		242		387		
Basic model	1.00	0.94	0.84, 1.05	0.92	0.82, 1.03	0.79	0.71, 0.89	0.83	0.73, 0.93	**6 x 10** ^**–5**^
Extended multivariable model	1.00	1.02	0.91, 1.14	1.05	0.94, 1.18	0.93	0.82, 1.05	0.99	0.87, 1.12	0.40
Excluding diet changers	1.00	1.06	0.94, 1.20	1.03	0.91, 1.17	0.93	0.81, 1.06	0.99	0.86, 1.15	0.38
Juice										
Cases/follow-up	1449/157978	523/69283		467/69356		482/68316				
Mean intakes (g/day)	0	11		87		235				
Basic model	1.00	0.89	0.81, 0.99	0.87	0.79, 0.97	0.93	0.84, 1.03			**0.03**
Extended multivariable model	1.00	0.98	0.88, 1.08	0.97	0.87, 1.08	0.99	0.89, 1.10			0.66
Excluding diet changers	1.00	1.01	0.90, 1.13	0.98	0.86, 1.10	0.99	0.88, 1.12			0.79
Potatoes										
Cases/follow-up	490/74186	544/73688		562/73110		620/72439		705/71511		
Mean intakes (g/day)	45	86		115		147		212		
Basic model	1.00	1.00	0.89, 1.13	0.97	0.86, 1.10	1.03	0.92, 1.17	1.14	1.01, 1.28	**0.02**
Extended multivariable model	1.00	0.99	0.88, 1.12	0.96	0.85, 1.09	0.97	0.86, 1.10	1.04	0.92, 1.17	0.60
Excluding diet changers	1.00	0.98	0.85, 1.13	0.98	0.85, 1.13	1.00	0.87, 1.14	1.07	0.94, 1.23	0.25
Whole grains										
Cases/follow-up	710/69847	551/72618		559/72959		514/74325		587/75184		
Mean intakes (portions/day)	0.0	0.3		0.7		1.2		2.5		
Basic model	1.00	0.82	0.74, 0.92	0.83	0.74, 0.92	0.70	0.63, 0.79	0.75	0.67, 0.83	**5 x 10** ^**–9**^
Extended multivariable model	1.00	0.89	0.80, 1.00	0.92	0.82, 1.02	0.80	0.72, 0.90	0.87	0.77, 0.97	**0.002**
Excluding diet changers	1.00	0.88	0.78, 1.00	0.89	0.79, 1.01	0.77	0.67, 0.87	0.81	0.71, 0.92	**0.0001**
Refined grains										
Cases/follow-up	564/73958	577/73228		598/72561		587/72359		595/72827		
Mean intakes (portions/day)	1.2	2.0		2.5		3.1		4.3		
Basic model	1.00	1.02	0.91, 1.14	1.04	0.93, 1.17	1.02	0.91, 1.15	1.02	0.91, 1.15	0.73
Extended multivariable model	1.00	1.03	0.92, 1.16	1.05	0.94, 1.18	1.05	0.94, 1.18	1.06	0.95, 1.20	0.28
Excluding diet changers	1.00	1.04	0.91, 1.19	1.07	0.93, 1.22	1.09	0.95, 1.25	1.09	0.95, 1.25	0.15
Cookies and cakes										
Cases/follow-up	610/71589	534/73455		568/73779		599/73431		610/72680		
Mean intakes (g/day)	6	20		33		49		79		
Basic model	1.00	0.85	0.76, 0.96	0.87	0.77, 0.97	0.84	0.75, 0.94	0.81	0.72, 0.91	**0.001**
Extended multivariable model	1.00	0.91	0.81, 1.03	0.96	0.85, 1.07	0.93	0.83, 1.05	0.90	0.80, 1.01	0.15
Excluding diet changers	1.00	0.90	0.79, 1.03	0.91	0.80, 1.04	0.89	0.78, 1.02	0.87	0.76, 1.00	0.07
Sugar and sweets										
Cases/follow-up	565/73201	579/73289		590/73494		583/72824		604/72125		
Mean intakes (g/day)	12	27		39		51		76		
Basic model	1.00	1.01	0.90, 1.13	1.00	0.89, 1.12	0.97	0.86, 1.09	1.10	0.98, 1.24	0.25
Extended multivariable model	1.00	1.03	0.91, 1.16	1.04	0.92, 1.16	0.98	0.87, 1.10	1.08	0.96, 1.21	0.46
Excluding diet changers	1.00	1.01	0.88, 1.16	1.07	0.94, 1.22	1.00	0.87, 1.15	1.09	0.95, 1.25	0.31
Sugar-sweetened beverages										
Cases/follow-up	1342/164894	490/67500		532/67072		557/65467				
Mean intakes (g/day)	0	26		89		309				
Basic model	1.00	0.89	0.80, 0.99	1.05	0.95, 1.16	1.04	0.94, 1.15			0.27
Extended multivariable model	1.00	0.93	0.84, 1.03	1.06	0.95, 1.17	1.00	0.90, 1.10			0.69
Excluding diet changers	1.00	0.95	0.85, 1.07	1.05	0.93, 1.17	0.99	0.88, 1.11			0.83

^a^Basic model adjusted for age, sex, season, diet method version and energy intake.

^b^Extended multivariable model adjusted for age, sex, season, diet method version, energy intake, BMI, smoking, alcohol consumption, leisure-time physical activity, and education.

^c^Extended multivariable adjustments were used in the model excluding diet changers.

### Effect modification by genetic risk for dyslipidemia

Genetic risk scores were composed of 26 SNPs for triglycerides, 41 SNPs for HDL-C and 32 SNPs for LDL-C (S4 Table) and were calculated for 24,799 individuals who had more than 60% of their SNPs successfully genotyped. The associations with genetic risk scores were strong as expected for their respective traits, triglycerides (p = 1x10^-48^), HDL-C (p = 7x10^-62^), and LDL-C (p = 2x10^-76^). The genetic risk scores for LDL-C and HDL-C were significantly associated with incidence of iCVD (p = 4x10^-6^ and 0.01, respectively), while the genetic risk score for triglycerides was not associated with iCVD (p = 0.13). Only one of the examined dietary variables showed nominal significant interaction with at least one of the genetic risk scores (Table 4). Specifically, higher potato consumption was associated with lower HDL-C concentrations among those with lower genetic risk for low HDL-C and not among those with higher genetic risk (p-interaction = 0.02). Only minor differences in the interactions were seen after adjusting for the other genetic risk scores.

**Table 4:** The association between diet intakes and triglycerides, HDL-C and LDL-C concentrations stratified by genetic risk scores.^a^

	GRS for triglycerides^b^		GRS for HDL-C		GRS for LDL-C	
	Low	High	Low	High	Low	High
Carbohydrates	0.012^c^(-0.004, 0.028)	0.023(0.003, 0.042)	-0.029(-0.040, -0.019)	-0.021(-0.030, -0.011)	-0.025(-0.053, 0.005)	<0.001(-0.031, 0.031)
	P-interaction = 0.82		P-interaction = 0.73		P-interaction = 0.48	
Sucrose	0.024(0.008, 0.040)	0.025(0.006, 0.045)	-0.030(-0.040, -0.020)	-0.017(-0.026, -0.008)	0.010(-0.019, 0.038)	0.008(-0.022, 0.038)
	P-interaction = 0.42		P-interaction = 0.52		P-interaction = 0.52	
Fiber	-0.004(-0.020, 0.012)	-0.016(-0.035, 0.004)	0.004(-0.006, 0.014)	-0.005(-0.014, 0.005)	-0.025(-0.054, 0.004)	0.005(-0.025, 0.036)
	P-interaction = 0.86		P-interaction = 0.66		P-interaction = 0.27	
Vegetables	-0.011(-0.026, 0.005)	-0.004(-0.023, 0.014)	0.005(-0.005, 0.014)	0.011(0.020, 0.020)	-0.002(-0.030, 0.026)	0.013(-0.016, 0.042)
	P-interaction = 0.93		P-interaction = 0.97		P-interaction = 0.89	
Fruits and berries	-0.005(-0.021, 0.011)	0.002(-0.021, 0.017)	0.001(-0.009, 0.012)	-0.004(-0.013, 0.005)	-0.023(-0.052, 0.005)	0.012(-0.019, 0.043)
	P-interaction = 0.60		P-interaction = 0.83		P-interaction = 0.54	
Juice	0.009(-0.010, 0.027)	-0.012(-0.034, 0.011)	<0.001(-0.012, 0.011)	-0.006(-0.017, 0.005)	0.014(-0.019, 0.047)	0.005(-0.030, 0.040)
	P-interaction = 0.40		P-interaction = 0.13		P-interaction = 0.63	
Potatoes	0.005(-0.011, 0.020)	0.010(-0.008, 0.029)	-0.011(-0.021, -0.001)	0.003(-0.006, 0.012)	-0.001(-0.029, 0.026)	-0.003(-0.032, 0.026)
	P-interaction = 0.24		P-interaction = **0.02**		P-interaction = 0.51	
Whole grains	-0.006(-0.021, 0.009)	-0.010(-0.028, 0.009)	0.008(-0.002, 0.018)	-0.006(-0.015, 0.003)	0.006(-0.020, 0.033)	<0.001(-0.030, 0.029)
	P-interaction = 0.54		P-interaction = 0.08		P-interaction = 0.82	
Refined grains	-0.002(-0.017, 0.013)	<0.001(-0.019, 0.018)	-0.002(-0.012, 0.008)	0.001(-0.007, 0.010)	-0.021(-0.049, 0.007)	0.005(-0.023, 0.033)
	P-interaction = 0.86		P-interaction = 0.65		P-interaction = 0.19	
Cookies and cakes	0.002(-0.014, 0.017)	0.003(-0.017, 0.022)	-0.014(-0.024, -0.003)	-0.005(-0.014, 0.004)	0.034(0.005, 0.063)	0.016(-0.014, 0.046)
	P-interaction = 0.91		P-interaction = 0.74		P-interaction = 0.87	
Sugar and sweets	0.017(0.001, 0.032)	0.016(-0.003, 0.035)	-0.017(-0.027, -0.007)	-0.013(-0.022, -0.004)	0.005(-0.023, 0.033)	-0.018(-0.048, 0.012)
	P-interaction = 0.41		P-interaction = 0.39		P-interaction = 0.93	
Sugar-sweetened beverages	0.028(0.009, 0.047)	0.039(0.016, 0.062)	-0.022(-0.035, -0.010)	-0.012(-0.023, -0.001)	0.026(-0.008, 0.059)	0.033(-0.003, 0.069)
	P-interaction = 0.75		P-interaction = 0.39		P-interaction = 0.51	

^a^Adjusted for age, sex, season, energy intake, BMI, smoking, alcohol consumption, leisure-time physical activity, education, and use of lipid-lowering therapies.

^b^Genetic risk scores were split by the median value. The interactions were examined with continuous variables of diet categories and genetic risk scores.

^c^Beta (β, regression coefficient) represents the mean change in biological risk marker for each increase in intake category.

We found no strong evidence of an interaction on iCVD risk between the dietary categories and genetic risk scores for standard lipids (Table 5). The strongest interaction on the risk of iCVD was observed between sugar and sweets consumption and genetic risk score for triglycerides (p-interaction = 0.05), where the intake was positively associated with iCVD risk only among individuals with a low genetic risk for high triglycerides.

**Table 5:** The association between diet intakes and incident cardiovascular disease stratified by genetic risk scores for high triglycerides, low HDL-C and high LDL-C.^a^

	GRS^b^ for triglycerides		GRS for HDL-C		GRS for LDL-C	
	Low	High	Low	High	Low	High
Carbohydrates	1.02 (0.98, 1.06)^c^	1.00 (0.96, 1.04)	0.99 (0.95, 1.04)	1.02 (0.98, 1.06)	1.04 (1.00, 1.08)	0.98 (0.94, 1.02)
	P-interaction = 0.11		P-interaction = 0.47		P-interaction = 0.75	
Sucrose	1.05 (1.01, 1.09)	0.99 (0.95, 1.03)	1.02 (0.98, 1.06)	1.02 (0.98, 1.06)	1.01 (0.97, 1.06)	1.02 (0.98, 1.06)
	P-interaction = 0.06 (0.06)		P-interaction = 0.74		P-interaction = 0.57	
Fiber	0.97 (0.94, 1.01)	0.96 (0.92, 1.00)	0.98 (0.94, 1.02)	0.96 (0.92, 1.00)	0.97 (0.93, 1.01)	0.96 (0.92, 1.00)
	P-interaction = 0.42		P-interaction = 0.34		P-interaction = 0.57	
Vegetables	1.00 (0.96, 1.04)	0.97 (0.93, 1.01)	0.99 (0.95, 1.03)	0.99 (0.95, 1.02)	0.99 (0.95, 1.04)	0.98 (0.94, 1.02)
	P-interaction = 0.26		P-interaction = 0.85		P-interaction = 0.50	
Fruits and berries	1.01 (0.97, 1.06)	0.96 (0.92, 1.00)	1.00 (0.96, 1.04)	0.98 (0.94, 1.02)	1.00 (0.96, 1.05)	0.97 (0.93, 1.01)
	P-interaction = 0.45		P-interaction = 0.61		P-interaction = 0.43	
Juice	0.99 (0.94, 1.04)	1.00 (0.96, 1.05)	1.00 (0.95, 1.05)	0.99 (0.94, 1.04)	1.02 (0.97, 1.07)	0.97 (0.93, 1.02)
	P-interaction = 0.61		P-interaction = 0.12		P-interaction = 0.39	
Potatoes	1.03 (0.99, 1.07)	1.00 (0.97, 1.04)	1.02 (0.98, 1.07)	1.00 (0.97, 1.04)	1.03 (0.99, 1.07)	1.00 (0.96, 1.04)
	P-interaction = 0.62		P-interaction = 0.08		P-interaction = 0.48	
Whole grains	0.96 (0.93, 1.00)	0.96 (0.92, 1.00)	0.97 (0.93, 1.00)	0.96 (0.92, 0.99)	0.96 (0.92, 0.99)	0.97 (0.93, 1.01)
	P-interaction = 0.64		P-interaction = 0.40		P-interaction = 0.21	
Refined grains	1.00 (0.97, 1.04)	1.03 (1.00, 1.07)	1.01 (0.97, 1.05)	1.03 (0.99, 1.07)	1.02 (0.98, 1.06)	1.02 (0.98, 1.06)
	P-interaction = 0.23		P-interaction = 0.55		P-interaction = 0.56	
Cookies and cakes	0.97 (0.93, 1.01)	1.00 (0.96, 1.04)	0.98 (0.94, 1.02)	0.99 (0.95, 1.03)	0.96 (0.92, 1.00)	1.01 (0.97, 1.05)
	P-interaction = 0.59		P-interaction = 0.56		P-interaction = 0.11	
Sugar and sweets	1.04 (1.00, 1.09)	0.98 (0.94, 1.02)	1.01 (0.97, 1.06)	1.01 (0.97, 1.05)	0.99 (0.95, 1.03)	1.03 (0.99, 1.07)
	P-interaction = 0.05 (0.050)		P-interaction = 0.14		P-interaction = 0.87	
Sugar- sweetened	1.01 (0.97, 1.06)	1.01 (0.97, 1.06)	1.00 (0.96, 1.05)	1.02 (0.98, 1.07)	1.01 (0.96, 1.06)	1.01 (0.97, 1.06)
beverages	P-interaction = 0.65		P-interaction = 0.38		P-interaction = 0.85	

^a^Adjusted for age, sex, season, diet method version, energy intake, BMI, smoking, alcohol consumption, leisure-time physical activity, and education.

^b^GRS, genetic risk score was split by the median value. The interactions were examined with continuous variables of diet categories and genetic risk scores.

^c^HR represents the risk estimate for each increase in intake category.

## Discussion

In this prospective cohort study that examined foods rich in carbohydrates, individuals who consumed a high amount of whole grains had a decreased risk of iCVD. Similarly, we observed a decreased risk of iCVD among individuals who consumed high levels of dietary fiber. None of the other foods showed a significant linear association with iCVD risk. We found no convincing evidence that genetic susceptibility to dyslipidemia, measured as the genetic risk scores of 80 validated genetic variants for blood lipids and lipoproteins, modified the association between carbohydrates and carbohydrate-rich foods and iCVD risk.

In line with our results, a high consumption of whole grains was found to be related to decreased CVD risk in several earlier studies [1, 2]. According to a meta-analysis with studies published up before February 2012, a consistent inverse association was observed across the 10 prospective cohort studies conducted in the US. The highest intake category of whole grains (on average 3–5 portions/day) was associated with a 21% reduction in CVD risk compared to those who rarely consumed whole grains. Because of the different sources of whole grains between countries [23, 24], additional studies are needed in other countries and ethnic groups. Interestingly, although whole grains were associated with decreased iCVD risk, we did not observe any association between consumption and any of the examined clinical risk factors at baseline. However, we found that higher fiber consumption was associated with lower levels of insulin resistance at baseline. Additionally, we did not observe the association between whole grain intake and risk of iCVD to be modified by the genetic risk scores for lipids and lipoproteins. The mechanism by which whole grain intake mediates a decreased CVD risk is not fully understood. Although whole grain interventions have been shown to have beneficial effects on blood levels of insulin, glucose and LDL-C [1], it is possible that the protective effects are mediated through other mechanisms, such as inflammation, oxidative stress, endothelial dysfunction [25], or an effect on gut microflora composition [26].

The consumption of sugar-sweetened beverages, as well as sugar and sweets, was related to higher triglyceride and lower HDL-C levels at baseline. Added sugar in the form of sucrose is most likely the main source of fructose in the Swedish diet [27]. In several recently published experimental studies, high fructose and sucrose consumption was related to adverse effects on blood lipids and lipoproteins. For example, an intervention study that compared the effects of very-high fructose and very-high glucose diets over 4 weeks found that triglyceride concentrations were adversely effected by fructose intake [28]. A 6-months study found that sucrose-sweetened soft drinks adversely affected triglycerides and total cholesterol levels more strongly compared to iso-caloric milk and non-caloric soft drinks [29]. However, the associations between sugar-sweetened beverage consumption and adverse effects on triglyceride and HDL-C levels in the MDCS cohort could not clearly be translated to an increased risk of iCVD; although, we have previously shown in the MDCS cohort that men who consumed less than 10% of their energy from sucrose had 14% lower risk of iCVD compared to men with higher sucrose intake levels [6]. However, we found a stronger association between high sugar and sweets consumption and iCVD risk among individuals with low genetic risk score for triglycerides.

Few earlier studies have examined the association between sugar and sugar-rich foods and CVD risk. Women in the US-based Nurses’ Health Study who consumed more than two sugar-sweetened beverages per day had a 35% higher risk of coronary heart disease compared with those who consumed less than one sugar-sweetened beverage per month [3]. In the Health Professionals Follow-Up Study that evaluated 42,883 men, each daily serving of sugar-sweetened beverages increased the men’s risk of coronary heart disease by 19%. In line with our study, high sugar-sweetened beverage consumption was associated with lower HDL-C and higher triglycerides levels [4]. High consumption of sugar-sweetened soda was also associated with an increased risk of stroke in the Nurses’ Health study and the Health Professional Follow-up Study [30]. However, data from populations outside the US are limited. The results from the Japan Public Health Centre-based study cohort suggest a positive association between soft drink intake and ischemic stroke [31]. The null association between sugar-sweetened beverages and iCVD risk in our study might, at least partly, be due to the relatively low intake levels and narrow intake range in our study population. However, an intake of 300 g/day (approximately 1 can per day) of sugar-sweetened beverages in the highest intake group is, although lower, still comparable to other studies. Another putative contributing factor for the null association could be the increase in the consumption of sugar-sweetened beverages in Sweden since 1990s, which may have weakened the value of this diet variable in the prospective analysis. In fact, cross-sectional analyses indicated an association between sugar-sweetened beverages consumption and adverse levels of triglycerides, HDL-C and HOMA-IR as well as high systolic blood pressure, which may support this possibility. It is also possible that consumption of sugar-sweetened beverages is an indicator an unhealthy lifestyle and that this phenomenon may be stronger in specific populations or age groups.

The relative validity of the diet method used in the MDCS is generally high, with energy-adjusted correlations of 0.60 among men and 0.74 among women for sugar. Although the dietary instrument used in the MDCS was not specifically constructed to estimate whole grain intake, it was specifically focused on estimating fiber intake in this middle-aged population. For example there was careful examination of fiber content in bread. The almost complete follow-up of individuals through registries and the detailed ascertainment and verification of CVD diagnosis are major strengths of this study. The estimated risks were clearly attenuated after adjusting for potential confounders, with smoking as the strongest factor affecting the risk estimates. However, we cannot exclude that residual confounding is still a problem. Another limitation might be the low participation rate of 40%. However, while the proportion reporting good health was higher in the MDCS than in a mailed health survey (where 74.6% participated), the socio-demographic structure, and the prevalence of obesity and smoking were similar [32]. In addition, for our research question, the internal validity is more important than having a representative study sample.

In this study, we accounted for genetic susceptibility to dyslipidemia by combining 80 validated genetic variants associated with blood lipids and lipoproteins. Together, these variants have been estimated to account for 25–30% of the genetic variance in blood lipid and lipoprotein levels in Caucasians and can be used as an estimate of the overall genetic susceptibility to dyslipidemia. However, these genetic variants are associated with different mechanisms of lipid and lipoprotein metabolism. By combining such different mechanisms, we may end up neutralizing the possible interacting effects due to the different directions of the interactions by the individual genetic markers. Thus, we found no convincing evidence that the combined genetic risk score of 80 common variants associated with dyslipidemia would modify the association between dietary intakes of carbohydrate-rich foods and iCVD risk, particularly if we corrected the p-values for multiple tests. Future studies may need to examine genetic variants affecting specific mechanisms and pathways separately, to address whether genetic susceptibility affecting specific mechanisms or pathways would modify the effect of dietary intakes on iCVD risk. Few earlier studies have combined individual SNPs into genetic risk scores and examined their interaction with lifestyle factors [10]. Pollin et al. found an attenuated lipid-lowering effect of a weight-loss intervention among those with high genetic risk score composed of 32 lipid-associated SNPs [10]. In addition, there are some studies examining interactions with individual genetic markers involved in lipid metabolism [33] [34] [35]; although, to our knowledge, no study has examined carbohydrate quality as a dietary exposure.

In conclusion, the results from the present study indicate that, among the food sources of carbohydrates, whole grain consumption affords the highest protection against iCVD. In addition, we found no convincing evidence that susceptibility to dyslipidemia, estimated as a genetic risk score of 80 validated genetic variants, modifies the association between these foods and iCVD risk.

## Supporting Information

S1 Table Baseline characteristics and dietary intakes according to highest and lowest category of energy-adjusted food intakes among 16,397 women and 10,048 men in the Malmö Diet and Cancer cohort. (DOC)

S2 Table Hazard ratios of incident cardiovascular events for the highest vs. lowest intake group in men and women, Malmö Diet and Cancer cohort, 1991–2009. (DOC)

S3 Table Hazard ratios of incident coronary event and stroke by categories of food groups, Malmö diet and cancer cohort, 1991–2009. (DOC)

S4 Table Characteristics of the included single nucleotide polymorphisms. (DOC)
