# Supplementary material for: The Association between Carbohydrate-Rich Foods and Risk of Cardiovascular Disease Is Not Modified by Genetic Susceptibility to Dyslipidemia as Determined by 80 Validated Variants
Source: PLoS One. 2015 Apr 21;10(4):e0126104. doi: 10.1371/journal.pone.0126104 (PMC4405383; doi:10.1371/journal.pone.0126104)
Supplement: S1 Table — (DOC) [file pone.0126104.s001.doc]

**S1 Table. Baseline characteristics and dietary intakes according to highest and lowest category of energy-adjusted food intakes among 16,397 women and 10,048 men in the Malmö Diet and Cancer cohort^1^**

|  | **Carbo-hydrate** | | **Sucrose** | | **Fiber** | | **Vegetables** | | **Fruit and berries** | | **Juice** | | **Potato** | | **Whole grains** | | **Refined grains** | | **Cookies and cakes** | | **Sugar and sweets** | | **Sugar-sweetened beverages** | |
| --- | --- | --- | --- | --- | --- | --- | --- | --- | --- | --- | --- | --- | --- | --- | --- | --- | --- | --- | --- | --- | --- | --- | --- | --- |
| Intake categories | **1** | **5** | **1** | **5** | **1** | **5** | **1** | **5** | **1** | **5** | **0** | **3** | **1** | **5** | **1** | **5** | **1** | **5** | **1** | **5** | **1** | **5** | **0** | **3** |
| **Women** |  |  |  |  |  |  |  |  |  |  |  |  |  |  |  |  |  |  |  |  |  |  |  |  |
| Age, y | 56.2  (7.5) | 58.1  (7.9) | 55.8  (7.4) | 57.7  (8.0) | 56.4  (7.9) | 57.7  (7.5) | 58.7  (8.1) | 55.7  (7.1) | 55.7  (7.7) | 57.7  (7.7) | 57.7  (7.8) | 57.5  (8.0) | 56.2  (7.7) | 57.9  (7.8) | 56.7  (8.2) | 58.2  (7.4) | 57.8  (7.4) | 56.1  (8.0) | 54.9  (7.4) | 60.0  (7.7) | 56.3  (7.6) | 57.6  (7.9) | 57.5  (7.8) | 57.1  (8.1) |
| BMI, kg/m^2^ | 25.0  (4.1) | 25.3  (4.1) | 25.4  (4.3) | 25.2  (4.2) | 25.0  (4.2) | 25.2  (4.1) | 25.4  (4.4) | 25.4  (4.1) | 24.9  (4.3) | 25.5  (4.1) | 25.5  (4.3) | 25.1  (4.1) | 25.0  (4.1) | 25.7  (4.4) | 25.3  (4.3) | 25.2  (4.1) | 25.4  (4.2) | 25.2  (4.2) | 25.2  (4.3) | 25.4  (4.1) | 25.7  (4.4) | 24.9  (4.0) | 25.3  (4.2) | 25.7  (4.4) |
| Protein, E% | 16.9  (2.9) | 15.0  (2.3) | 17.7  (2.8) | 14.3  (2.1) | 15.5  (2.7) | 16.2  (2.7) | 15.2  (2.5) | 16.7  (2.8) | 15.9  (2.7) | 15.9  (2.6) | 16.1  (2.7) | 15.6  (2.3) | 15.8  (2.7) | 16.4  (2.5) | 15.4  (2.6) | 16.2  (2.6) | 16.4  (3.0) | 15.8  (2.3) | 17.0  (3.0) | 15.0  (2.2) | 17.4  (2.8) | 14.9  (2.3) | 16.5  (2.7) | 14.9  (2.3) |
| Fat, E% | 45.8  (4.4) | 31.5  (3.9) | 41.4  (6.5) | 36.5  (5.7) | 42.9  (5.8) | 34.2  (5.5) | 40.0  (6.4) | 36.9  (6.0) | 42.1  (5.9) | 35.4  (5.7) | 39.1  (6.2) | 37.0  (5.7) | 38.5  (6.3) | 38.1  (5.9) | 40.1  (6.1) | 36.2  (6.0) | 39.2  (6.5) | 36.5  (5.7) | 38.3  (7.1) | 38.6  (5.4) | 38.8  (6.8) | 37.5  (5.7) | 38.7  (6.3) | 37.3  (5.7) |
| Carbohydrates, E% | 37.3  (3.3) | 53.5  (3.5) | 40.9  (6.0) | 49.2  (5.4) | 41.6  (5.9) | 49.6  (5.5) | 44.8  (6.3) | 46.4  (6.1) | 42.0  (5.9) | 48.7  (5.7) | 44.9  (6.1) | 47.4  (5.7) | 45.8  (6.3) | 45.5  (5.9) | 44.5  (6.1) | 47.6  (5.9) | 44.4  (6.5) | 47.8  (5.7) | 44.7  (7.2) | 46.4  (5.3) | 43.8  (6.6) | 47.6  (5.6) | 44.8  (6.1) | 47.8  (5.8) |
| Sucrose, E% | 6.4  (2.3) | 11.4  (4.0) | 4.5  (1.0) | 13.7  (2.8) | 9.5  (4.4) | 8.6  (2.8) | 9.5  (4.0) | 8.4  (2.9) | 8.4  (4.1) | 9.4  (3.0) | 8.6  (3.6) | 9.5  (3.3) | 9.3  (3.5) | 8.0  (3.2) | 9.5  (4.0) | 8.2  (2.9) | 9.3  (3.8) | 7.8  (3.0) | 7.6  (3.8) | 9.9  (3.1) | 6.5  (2.6) | 11.5  (3.8) | 7.7  (2.9) | 11.8  (3.6) |
| Fiber, g/1000 kcal | 7.9  (2.2) | 11.2  (3.2) | 9.4  (2.9) | 8.9  (2.7) | 6.2  (0.9) | 13.3  (2.3) | 7.8  (2.3) | 11.5  (3.0) | 7.2  (2.0) | 11.7  (2.8) | 9.6  (2.9) | 9.4  (2.6) | 9.8  (3.0) | 9.5  (2.8) | 7.8  (2.2) | 11.9  (2.9) | 10.1  (3.2) | 9.7  (2.5) | 9.9  (3.5) | 9.3  (2.4) | 10.4  (3.2) | 8.8  (2.5) | 10.0  (2.9) | 8.8  (2.5) |
| Vegetables, g/d | 176  (94) | 195  (111) | 194  (111) | 168  (90) | 117  (56) | 263  (126) | 72  (22) | 328  (94) | 137  (77) | 233  (118) | 180  (102) | 190  (98) | 203  (112) | 169  (94) | 160  (93) | 210  (112) | 203  (110) | 168  (92) | 206  (122) | 162  (84) | 220  (122) | 158  (84) | 194  (107) | 173  (91) |
| Fruits and berries, g/d | 143  (92) | 276  (150) | 157  (103) | 218  (135) | 99  (62) | 317  (142) | 152  (107) | 267  (135) | 53  (25) | 379  (104) | 202  (127) | 215  (125) | 230  (137) | 182  (116) | 176  (124) | 238  (126) | 232  (139) | 179  (115) | 208  (145) | 201  (110) | 226  (140) | 182  (113) | 212  (127) | 197  (124) |
| Whole grain, port/d | 0.71  (0.65) | 1.16  (1.07) | 1.01  (1.00) | 0.76  (0.76) | 0.35  (0.38) | 1.65  (1.15) | 0.72  (0.82) | 1.11  (0.93) | 0.66  (0.76) | 1.09  (0.93) | 0.91  (0.87) | 0.92  (0.85) | 1.01  (0.95) | 0.77  (0.78) | 0.05  (0.06) | 2.28  (0.87) | 1.46  (1.08) | 0.51  (0.58) | 0.98  (0.99) | 0.82  (0.75) | 0.98  (0.96) | 0.78  (0.77) | 0.96  (0.89) | 0.79  (0.79) |
| Fish, g/d | 41.4  (32.0) | 34.6  (27.0) | 39.0  (31.0) | 33.8  (26.4) | 32.9  (28.2) | 42.5  (30.6) | 31.3  (26.3) | 44.0  (31.9) | 31.5  (27.0) | 41.6  (30.5) | 35.4  (29.2) | 38.4  (28.0) | 33.1  (27.9) | 41.6  (30.3) | 32.8  (28.0) | 40.7  (30.1) | 44.1  (31.4) | 30.4  (25.2 | 37.8  (31.5) | 36.3  (26.8) | 41.0  (32.4) | 32.6  (25.6) | 38.8  (29.3) | 35.0  (28.2) |
| Meat, g/d | 128  (54) | 94  (43) | 121  (53) | 107  (46) | 121  (51) | 100  (50) | 107  (45) | 113  (54) | 120  (51) | 106  (50) | 112  (49) | 110  (47) | 101  (49) | 123  (50) | 120  (50) | 105  (48) | 116  (53) | 103  (46) | 116  (56) | 105  (41) | 116  (53) | 105  (44) | 108  (48) | 115  (50) |
| **Men** |  |  |  |  |  |  |  |  |  |  |  |  |  |  |  |  |  |  |  |  |  |  |  |  |
| Age, y | 58.2  (6.8) | 59.2  (7.1) | 58.0  (6.7) | 59.3  (7.2) | 58.6  (7.1) | 59.1  (7.0) | 59.8  (7.1) | 57.7  (6.7) | 58.0  (6.9) | 59.0  (6.9) | 59.3  (6.9) | 58.6  (6.9) | 58.0  (7.0) | 59.1  (6.8) | 58.7  (7.1) | 59.4  (7.0) | 59.2  (6.8) | 58.4  (7.0) | 57.3  (6.6) | 61.7  (6.9) | 57.6  (6.7) | 60.1  (7.2) | 59.3  (7.0) | 58.6  (7.0) |
| BMI, kg/m^2^ | 26.4  (3.6) | 25.8  (3.3) | 26.4  (3.6) | 25.8  (3.4) | 26.1  (3.6) | 26.0  (3.4) | 26.1  (3.5) | 26.3  (3.5) | 25.8  (3.4) | 26.7  (3.5) | 26.2  (3.4) | 26.2  (3.4) | 26.2  (3.6) | 26.2  (3.4) | 26.2  (3.5) | 26.0  (3.4) | 26.5  (3.6) | 25.9  (3.4) | 26.3  (3.6) | 26.0  (3.3) | 26.6  (3.7) | 25.8  (3.3) | 26.0  (3.4) | 26.6  (3.6) |
| Protein, E% | 16.3  (2.7) | 14.6  (2.2) | 16.7  (2.6) | 13.9  (2.0) | 15.4  (2.6) | 15.6  (2.4) | 14.9  (2.4) | 16.1  (2.6) | 15.6  (2.6) | 15.2  (2.5) | 15.4  (2.5) | 15.3  (2.4) | 15.1  (2.7) | 15.9  (2.4) | 15.2  (2.5) | 15.7  (2.5) | 16.0  (2.8) | 15.3  (2.3) | 16.3  (2.8) | 14.5  (2.1) | 16.5  (2.6) | 14.3  (2.2) | 15.9  (2.6) | 14.6  (2.3) |
| Fat, E% | 46.5  (4.6) | 32.4  (4.2) | 41.9  (6.5) | 37.5  (5.9) | 43.8  (5.9) | 34.5  (5.6) | 41.1  (6.3) | 38.2  (6.3) | 42.3  (6.1) | 36.3  (6.1) | 40.2  (6.3) | 37.8  (6.1) | 39.8  (6.9) | 39.4  (5.9) | 41.2  (6.3) | 37.2  (6.1) | 40.9  (6.8) | 37.3  (5.8) | 40.0  (7.0) | 39.5  (5.3) | 40.9  (6.7) | 37.7  (5.8) | 40.3  (6.4) | 38.4  (6.0) |
| Carbohydrates, E% | 37.2  (3.7) | 53.1  (3.7) | 41.4  (6.0) | 48.7  (5.7) | 40.8  (6.0) | 49.9  (5.7) | 44.1  (6.2) | 45.7  (6.5) | 42.1  (6.0) | 48.5  (6.0) | 44.3  (6.3) | 46.9  (6.0) | 45.0  (6.7) | 44.7  (6.0) | 43.7  (6.3) | 47.1  (6.0) | 43.1  (6.9) | 47.4  (5.7) | 43.7  (6.9) | 46.1  (5,1) | 42.7  (6.6) | 48.0  (5.7) | 43.8  (6.3) | 47.1  (6.0) |
| Sucrose, E% | 5.9  (2.5) | 10.8  (4.5) | 4.4  (1.1) | 14.3  (3.1) | 8.8  (4.6) | 7.7  (2.9) | 8.7  (4.1) | 7.7  (3.3) | 7.8  (4.1) | 9.0  (3.4) | 8.1  (3.9) | 8.8  (3.4) | 8.7  (4.2) | 7.5  (3.6) | 8.6  (4.2) | 7.6  (3.2) | 8.7  (4.3) | 7.3  (3.2) | 7.2  (4.0) | 9.6  (3.5) | 5.6  (2.7) | 12.0  (4.3) | 7.0  (3.2) | 11.1  (3.8) |
| Fiber, g/1000 kcal | 6.6  (1.7) | 10.1  (3.0) | 8.2  (2.5) | 7.6  (2.4) | 5.6  (0.9) | 12.2  (2.3) | 7.0  (2.0) | 10.1  (2.9) | 6.8  (1.9) | 10.6  (2.8) | 8.2  (2.5) | 8.5  (2.5) | 8.4  (2.8) | 8.1  (2.4) | 6.9  (1.9) | 10.6  (2.7) | 8.5  (3.1) | 8.7  (2.2) | 8.1  (2.9) | 8.3  (2.1) | 8.6  (2.9) | 7.7  (2.4) | 8.6  (2.7) | 7.8  (2.2) |
| Vegetables, g/d | 162  (93) | 182  (111) | 176  (100) | 152  (90) | 118  (62) | 247  (131) | 74  (25) | 341  (95) | 141  (81) | 223  (125) | 166  (124) | 182  (101) | 189  (114) | 162  (92) | 153  (95) | 193  (108) | 191  (115) | 162  (93) | 180  (108) | 156  (88) | 194  (113) | 140  (83) | 174  (102) | 165  (96) |
| Fruit and berries, g/d | 119  (91) | 242  (168) | 140  (102) | 182  (134) | 90  (66) | 285  (178) | 137  (110) | 236  (158) | 54  (27) | 411  (137) | 166  (124) | 195  (139) | 198  (149) | 154  (112) | 148  (124) | 203  (144) | 198  (157) | 158  (114) | 165  (136) | 172  (114) | 182  (148) | 152  (109) | 172  (134) | 174  (130) |
| Whole grain, port/d | 0.72  (0.79) | 1.47  (1.61) | 1.14  (1.38) | 0.82  (0.97) | 0.35  (0.43) | 2.37  (1.76) | 0.85  (1.06) | 1.32  (1.34) | 0.79  (1.06) | 1.35  (1.37) | 1.03  (1.20) | 1.12  (1.21) | 1.17  (1.29) | 0.93  (1.09) | 0.05  (0.07) | 2.86  (1.29) | 1.86  (1.67) | 0.64  (0.80) | 1.05  (1.28) | 0.92  (1.01) | 1.16  (1.34) | 0.87  (0.97) | 1.11  (1.27) | 0.93  (1.08) |
| Fish, g/d | 50.2  (41.7) | 40.4  (37.0) | 50  (45) | 39  (34) | 41.7  (36.6) | 50.0  (44.7) | 39.6  (36.0) | 53.5  (45.1) | 40.9  (37.5) | 50.8  (43.4) | 42.6  (37.7) | 44.7  (38.0) | 38.1  (38.9) | 49.6  (41.4) | 40.9  (40.3) | 50.0  (42.1) | 54.1  (44.7) | 37.9  (36.2) | 47.2  (44.7) | 41.6  (33.2) | 50.6  (45.3) | 36.1  (29.8) | 47.0  (40.7) | 41.1  (37.1) |
| Meat, g/d | 192  (81) | 138  (58) | 179  (78) | 152  (62) | 175  (74) | 145  (69) | 161  (69) | 171  (80) | 176  (75) | 155  (69) | 169  (73) | 158  (69) | 149  (71) | 179  (77) | 177  (77) | 154  (68) | 176  (82) | 153  (64) | 180  (83) | 144  (55) | 181  (81) | 142  (56) | 161  (72) | 168  (70) |

^1^All vales are expressed as mean (SE) and the majority of differences in mean were statistically significant (*P*<0.05) due to the large sample size and therefore not shown.
