# Supplementary material for: The Association between Carbohydrate-Rich Foods and Risk of Cardiovascular Disease Is Not Modified by Genetic Susceptibility to Dyslipidemia as Determined by 80 Validated Variants
Source: PLoS One. 2015 Apr 21;10(4):e0126104. doi: 10.1371/journal.pone.0126104 (PMC4405383; doi:10.1371/journal.pone.0126104)
Supplement: S2 Table — (DOC) [file pone.0126104.s002.doc]

**S2 Table. Hazard ratios of incident cardiovascular events for the highest vs. lowest intake group in men and women, Malmö Diet and Cancer cohort, 1991-2009^1^**

|  | iCVD | | CHD | | Stroke | |
| --- | --- | --- | --- | --- | --- | --- |
|  | Men | Women | Men | Women | Men | Women |
| Carbohydrates |  |  |  |  |  |  |
| Q5 vs. Q1 | 0.93 (0.79-1.10) | 1.09 (0.90-1.32) | 0.99 (0.81-1.21) | 1.13 (0.86-1.48) | 0.84 (0.84-1.10) | 1.05 (0.79-1.38) |
| P-trend | 0.83 | 0.38 | 0.73 | 0.28 | 0.40 | 0.89 |
| Sucrose |  |  |  |  |  |  |
| Q5 vs. Q1 | 1.13 (0.97-1.31) | 1.00 (0.83-1.21) | 1.16 (0.97-1.39) | 1.02 (0.78-1.34) | 1.07 (0.83-1.37) | 0.98 (0.75-1.28) |
| P-trend | 0.07 | 0.97 | 0.17 | 0.71 | 0.24 | 0.66 |
| Fiber |  |  |  |  |  |  |
| Q5 vs. Q1 | 0.88 (0.75-1.04) | 0.84 (0.70-1.01) | 0.95 (0.78-1.17) | 0.82 (0.64-1.05) | 0.77 (0.59-1.01) | 0.86 (0.66-1.13) |
| P-trend | 0.08 | **0.05** | 0.65 | 0.11 | **0.02** | 0.25 |
| Vegetables |  |  |  |  |  |  |
| Q5 vs. Q1 | 0.92 (0.78-1.08) | 0.97 (0.80-1.17) | 0.89 (0.73-1.09) | 1.22 (0.93-1.59) | 0.97 (0.74-1.26) | 0.76 (0.58-1.00) |
| P-trend | 0.06 | 0.73 | 0.13 | **0.05** | 0.23 | 0.13 |
| Fruit and berries |  |  |  |  |  |  |
| Q5 vs. Q1 | 0.95 (0.81-1.13) | 0.99 (0.81-1.20) | 1.04 (0.85-1.28) | 0.91 (0.69-1.19) | 0.80 (0.60-1.07) | 1.07 (0.81-1.42) |
| P-trend | 0.43 | 0.64 | 0.97 | 0.19 | 0.16 | 0.49 |
| Juice |  |  |  |  |  |  |
| T3 vs. 0-consumers | 1.03 (0.89-1.20) | 0.93 (0.80-1.08) | 1.05 (0.87-1.27) | 0.94 (0.77-1.15) | 1.00 (0.78-1.28) | 0.92 (0.74-1.14) |
| P-trend | 0.90 | 0.45 | 0.90 | 0.41 | 0.72 | 0.82 |
| Potato |  |  |  |  |  |  |
| Q5 vs. Q1 | 1.03 (0.88-1.21) | 1.07 (0.90-1.28) | 1.03 (0.84-1.26) | 1.01 (0.79-1.29) | 1.04 (0.79-1.36) | 1.15 (0.88-1.49) |
| P-trend | 0.85 | 0.29 | 0.96 | 0.79 | 0.70 | 0.21 |
| Refined grains |  |  |  |  |  |  |
| Q5 vs. Q1 | 1.04 (0.89-1.22) | 1.12 (0.93-1.34) | 1.09 (0.89-1.32) | 1.12 (0.87-1.44) | 0.97 (0.74-1.26) | 1.12 (0.86-1.46) |
| P-trend | 0.59 | 0.32 | 0.29 | 0.39 | 0.62 | 0.61 |
| Whole grains |  |  |  |  |  |  |
| Q5 vs. Q1 | 0.92 (0.80-1.06) | 0.79 (0.65-0.94) | 0.96 (0.80-1.15) | 0.73 (0.57-0.93) | 0.84 (0.67-1.07) | 0.85 (0.66-1.12) |
| P-trend | 0.13 | **0.002** | 1.00 | **0.005** | **0.01** | 0.14 |
| Cookies and cakes |  |  |  |  |  |  |
| Q5 vs. Q1 | 0.93 (0.79-1.09) | 0.85 (0.70-1.02) | 0.95 (0.78-1.16) | 0.91 (0.70-1.19) | 0.90 (0.69-1.17) | 0.78 (0.61-1.01) |
| P-trend | 0.83 | 0.07 | 0.69 | 0.25 | 0.85 | 0.16 |
| Sugar and sweets |  |  |  |  |  |  |
| Q5 vs. Q1 | 1.14 (0.98-1.34) | 1.03 (0.86-1.24) | 1.10 (0.90-1.34) | 1.02 (0.79-1.32) | 1.22 (0.94-1.59) | 1.04 (0.80-1.36) |
| P-trend | 0.41 | 0.76 | 0.59 | 0.81 | 0.51 | 0.49 |
| Sugar-sweetened beverages |  |  |  |  |  |  |
| T3 vs. 0-consumers | 1.02 (0.90-1.16) | 0.96 (0.81-1.13) | 1.05 (0.90-1.24) | 0.95 (0.76-1.19) | 0.96 (0.78-1.20) | 0.97 (0.77-1.22) |
| P-trend | 0.64 | 0.98 | 0.55 | 0.98 | 0.97 | 0.99 |

^1^Adjusted for age, season, diet method version, energy intake, BMI, smoking, alcohol consumption, leisure-time physical activity, education
