# Supplementary material for: The Association between Carbohydrate-Rich Foods and Risk of Cardiovascular Disease Is Not Modified by Genetic Susceptibility to Dyslipidemia as Determined by 80 Validated Variants
Source: PLoS One. 2015 Apr 21;10(4):e0126104. doi: 10.1371/journal.pone.0126104 (PMC4405383; doi:10.1371/journal.pone.0126104)
Supplement: S3 Table — (DOC) [file pone.0126104.s003.doc]

**S3 Table. Hazard ratios of incident coronary event and stroke by categories of food groups, Malmö diet and cancer cohort, 1991-2009**^1^

|  | **Categories of intake** | | | | | | | | |  |
| --- | --- | --- | --- | --- | --- | --- | --- | --- | --- | --- |
| Food groups | **1** | **2** | | **3** | | **4** | | **5** | | **P_trend_** |
| Carbohydrates |  |  |  |  |  |  |  |  |  |  |
| Coronary event | 1.00 | 0.95 | 0.82-1.11 | 1.02 | 0.87-1.19 | 1.05 | 0.90-1.23 | 1.04 | 0.89-1.23 | 0.34 |
| Stroke | 1.00 | 0.92 | 0.77-1.11 | 0.93 | 0.77-1-12 | 0.94 | 0.78-1.14 | 0.94 | 0.77-1.14 | 0.66 |
| Sucrose |  |  |  |  |  |  |  |  |  |  |
| Coronary event | 1.00 | 0.95 | 0.81-1.10 | 1.01 | 0.87-1.18 | 0.97 | 0.83-1.13 | 1.11 | 0.95-1.28 | 0.19 |
| Stroke | 1.00 | 0.87 | 0.72-1.05 | 1.02 | 0.86-1.23 | 0.90 | 0.75-1.09 | 1.04 | 0.87-1.25 | 0.59 |
| Fiber |  |  |  |  |  |  |  |  |  |  |
| Coronary event | 1.00 | 0.96 | 0.83-1.10 | 0.91 | 0.79-1.06 | 0.89 | 0.76-1.04 | 0.92 | 0.79-1.08 | 0.18 |
| Stroke | 1.00 | 0.84 | 0.71-1.00 | 0.88 | 0.73-1.04 | 0.77 | 0.64-0.93 | 0.81 | 0.67-0.98 | **0.02** |
| Vegetables |  |  |  |  |  |  |  |  |  |  |
| Coronary event | 1.00 | 0.95 | 0.83-1.10 | 0.99 | 0.86-1.15 | 0.98 | 0.85-1.14 | 0.99 | 0.85-1.16 | 1.00 |
| Stroke | 1.00 | 0.99 | 0.84-1.16 | 0.85 | 0.71-1.01 | 0.90 | 0.76-1.08 | 0.86 | 0.71-1.04 | 0.06 |
| Fruit and berries |  |  |  |  |  |  |  |  |  |  |
| Coronary event | 1.00 | 1.06 | 0.92-1.22 | 1.09 | 0.94-1.26 | 0.91 | 0.78-1.07 | 1.00 | 0.85-1.18 | 0.49 |
| Stroke | 1.00 | 0.97 | 0.81-1.15 | 1.00 | 0.84-1.19 | 0.94 | 0.78-1.13 | 0.96 | 0.79-1.16 | 0.63 |
| Juice |  |  |  |  |  |  |  |  |  |  |
| Coronary event | 1.00 | 0.97 | 0.85-1.11 | 0.91 | 0.79-1.05 | 1.02 | 0.89-1.17 |  |  | 0.77 |
| Stroke | 1.00 | 0.99 | 0.85-1.16 | 1.04 | 0.89-1.23 | 0.95 | 0.80-1.12 |  |  | 0.73 |
| Potato |  |  |  |  |  |  |  |  |  |  |
| Coronary event | 1.00 | 0.95 | 0.80-1.11 | 0.91 | 0.78-1.07 | 0.94 | 0.80-1.09 | 1.01 | 0.86-1.17 | 0.84 |
| Stroke | 1.00 | 1.06 | 0.88-1.29 | 1.03 | 0.86-1.25 | 1.03 | 0.85-1.25 | 1.08 | 0.90-1.31 | 0.56 |
| Whole grains |  |  |  |  |  |  |  |  |  |  |
| Coronary event | 1.00 | 0.85 | 0.73-0.98 | 0.91 | 0.79-1.05 | 0.85 | 0.73-0.98 | 0.88 | 0.76-1.01 | 0.10 |
| Stroke | 1.00 | 0.96 | 0.81-1.14 | 0.92 | 0.78-1.10 | 0.75 | 0.62-0.90 | 0.85 | 0.71-1.01 | **0.006** |
| Refined grains |  |  |  |  |  |  |  |  |  |  |
| Coronary event | 1.00 | 0.98 | 0.85-1.15 | 1.04 | 0.89-1.21 | 1.08 | 0.92-1.25 | 1.08 | 0.93-1.26 | 0.17 |
| Stroke | 1.00 | 1.09 | 0.91-1.30 | 1.07 | 0.89-1.27 | 1.02 | 0.85-1.23 | 1.03 | 0.86-1.25 | 1.00 |
| Cookies and cakes |  |  |  |  |  |  |  |  |  |  |
| Coronary event | 1.00 | 1.02 | 0.88-1.18 | 1.02 | 0.87-1.18 | 0.97 | 0.83-1.13 | 0.93 | 0.79-1.09 | 0.28 |
| Stroke | 1.00 | 0.77 | 0.64-0.93 | 0.87 | 0.73-1.05 | 0.88 | 0.74-1.05 | 0.85 | 0.71-1.02 | 0.35 |
| Sugar and sweets |  |  |  |  |  |  |  |  |  |  |
| Coronary event | 1.00 | 1.03 | 0.88-1.20 | 1.09 | 0.94-1.27 | 0.96 | 0.82-1.12 | 1.06 | 0.90-1.24 | 0.82 |
| Stroke | 1.00 | 1.03 | 0.86-1.24 | 0.96 | 0.80-1.15 | 1.01 | 0.84-1.21 | 1.11 | 0.92-1.33 | 0.37 |
| Sugar-sweetened beverages |  |  |  |  |  |  |  |  |  |  |
| Coronary event | 1.00 | 0.98 | 0.85-1.12 | 1.05 | 0.92-1.20 | 1.02 | 0.89-1.16 |  |  | 0.59 |
| Stroke | 1.00 | 0.87 | 0.74-1.02 | 1.06 | 0.91-1.24 | 0.97 | 0.83-1.13 |  |  | 1.00 |

^1^Adjusted for age, sex, season, diet method version, energy intake, BMI, smoking, alcohol consumption, leisure-time physical activity, education
