# Supplementary material for: The Association between Carbohydrate-Rich Foods and Risk of Cardiovascular Disease Is Not Modified by Genetic Susceptibility to Dyslipidemia as Determined by 80 Validated Variants
Source: PLoS One. 2015 Apr 21;10(4):e0126104. doi: 10.1371/journal.pone.0126104 (PMC4405383; doi:10.1371/journal.pone.0126104)
Supplement: S4 Table — (DOC) [file pone.0126104.s004.doc]

**S4 Table. Characteristics of the included single nucleotide polymorphisms**

| **Chromosome** | **Locus** | **SNP** | **Alleles^1^/MAF** | **Lead trait/additional traits (effect size)** |
| --- | --- | --- | --- | --- |
| 1 | *LDLRAP1* | rs12027135 | T/A/0.45 | LDL (-1.10) |
| 1 | *PABPC4* | rs4660293 | A/G/0.25 | HDL (-0.48) |
| 1 | *PCSK9* | rs2479409 | A/G/0.35 | LDL (2.01) |
| 1 | *SORT1* | rs629301 | A/C/0.23 | LDL (-5.56) |
| 1 | *ZNF648* | rs1689800 | T/C/0.34 | HDL (-0.47) |
| 1 | *GALNT2* | rs4846914 | A/G/0.40 | HDL (-0.61)/TG (2.76) |
| 1 | *IRF2BP2* | rs514230 | A/T/0.48 | LDL (-1.13) |
| 2 | *APOB* | rs1042034 | A/G/0.20 | TG (-5.99)/HDL (0.90) |
| 2 | *APOB* | rs1367117 | G/A/0.34 | LDL (4.05) |
| 2 | *GCKR* | rs1260326 | C/T/0.37 | TG (8.76) |
| 2 | *ABCG5/8* | rs4299376 | T/G/0.29 | LDL (2.75) |
| 2 | *COBLL1* | rs12328675 | T/C/0.12 | HDL (0.68) |
| 2 | *IRS1* | rs2972146 | A/C/0.37 | HDL (0.46)/TG (-1.89) |
| 3 | *MSL2L1* | rs645040 | T/G/0.22 | TG (-2.22) |
| 4 | *KLHL8* | rs442177 | A/C/0.43 | TG (-2.25) |
| 4 | *SLC39A8* | rs13107325 | C/T/0.05 | HDL (-0.84) |
| 5 | *ARL15* | rs6450176 | G/A/0.25 | HDL (-0.49) |
| 5 | *MAP3K1* | rs9686661 | C/T/0.16 | TG (2.57) |
| 5 | *HMGCR* | rs12916 | T/C/0.41 | LDL (2.45) |
| 5 | *TIMD4* | rs6882076 | C/T/0.36 | LDL (-1.67)/TG (-2.63) |
| 6 | *MYLIP* | rs3757354 | G/A/0.24 | LDL (-1.43) |
| 6 | *HFE* | rs1800562 | G/A/0.05 | LDL (-2.22) |
| 6 | *HLA* | rs3177928 | G/A/0.13 | LDL (1.83) |
| 6 | *HLA* | rs2247056 | G/A/0.27 | TG (-2.99) |
| 6 | *C6orf106* | rs2814944 | G/A/0.16 | HDL (-0.49) |
| 6 | *FRK* | rs9488822 | A/T/0.31 | LDL (-0.89) |
| 6 | *CITED2* | rs605066 | T/C/0.44 | HDL (-0.39) |
| 6 | *LPA* | rs1564348 | A/G/0.15 | LDL (1.95) |
| 7 | *DNAH11* | rs12670798 | T/C/0.25 | LDL (1.26) |
| 7 | *MLXIPL* | rs17145738 | C/T/0.12 | TG (-9.32)/HDL (0.57) |
| 8 | *PPP1R3B* | rs9987289 | G/A/0.10 | HDL (-1.21)/LDL (-2.22) |
| 8 | *PINX1* | rs11776767 | G/C/0.34 | TG (2.01) |
| 8 | *NAT2* | rs1495741 | A/G/0.22 | TG (2.85) |
| 8 | *LPL* | rs12678919 | A/G/0.09 | TG (-13.64)/HDL (2.25) |
| 8 | *CYP7A1* | rs2081687 | C/T/0.34 | LDL (0.95) |
| 8 | *TRPS1* | rs2293889 | G/T/0.43 | HDL (-0.44) |
| 8 | *TRIB1* | rs2954029 | A/T/0.47 | TG (-5.64)/LDL (-1.84)/HDL (0.61) |
| 9 | *TTC39B* | rs581080 | C/G/0.19 | HDL (-0.65) |
| 9 | *ABCA1* | rs1883025 | G/A/0.24 | HDL (-0.94) |
| 9 | *ABO* | rs9411489 | G/A/0.21 | LDL (2.24) |
| 10 | *CYP26A1* | rs2068888 | G/A/0.44 | TG (-2.28) |
| 10 | *GPAM* | rs2255141 | G/A/0.29 | LDL (1.08) |
| 11 | *AMPD3* | rs2923084 | A/G/0.17 | HDL (-0.41) |
| 11 | *LRP4* | rs3136441 | T/C/0.14 | HDL (0.78) |
| 11 | *FADS123* | rs174546 | C/T/0.33 | TG (3.82)/HDL (-0.73)/LDL (-1.71) |
| 11 | *APOA1* | rs964184 | C/G/0.13 | TG (16.95)/HDL (-1.50)/LDL (2.85) |
| 11 | *UBASH3B* | rs7941030 | T/C/0.39 | HDL (0.31) |
| 11 | *ST3GAL4* | rs11220462 | C/T/0.14 | LDL (1.95) |
| 12 | *PDE3A* | rs7134375 | C/A/0.43 | HDL (0.40) |
| 12 | *LRP1* | rs11613352 | C/T/0.27 | TG (-2.70)/HDL (0.46) |
| 12 | *MVK* | rs7134594 | T/C/0.46 | HDL (-0.44) |
| 12 | *BRAP* | rs11065987 | A/G/0.42 | LDL (-0.97) |
| 12 | *HNF1A* | rs1169288 | T/G/0.32 | LDL (1.42) |
| 12 | *SBNO1* | rs4759375 | C/T/0.09 | HDL (0.86) |
| 12 | *ZNF664* | rs4765127 | G/T/0.33 | HDL (0.44)/TG (-2.42) |
| 14 | *NYNRIN* | rs8017377 | G/A/0.46 | LDL (1.14) |
| 15 | *CAPN3* | rs2412710 | G/A/0.02 | TG (7.00) |
| 15 | *FRMD5* | rs2929282 | A/T/0.04 | TG (5.13) |
| 15 | *LIPC* | rs1532085 | G/A/0.38 | HDL (1.45)/TG (2.99) |
| 15 | *LACTB* | rs2652834 | C/T/0.21 | HDL (-0.39) |
| 16 | *CTF1* | rs11649653 | C/G/0.41 | TG (-2.13) |
| 16 | *CETP* | rs3764261 | G/T/0.33 | HDL (3.39)/LDL (-1.45)/TG (-2.88) |
| 16 | *LCAT* | rs16942887 | G/A/0.14 | HDL (1.27) |
| 16 | *HPR* | rs2000999 | G/A/0.22 | LDL (2.00) |
| 16 | *CMIP* | rs2925979 | G/A/0.30 | HDL (-0.45) |
| 17 | *STARD3* | rs11869286 | C/G/0.32 | HDL (-0.48) |
| 17 | *PGS1* | rs4129767 | T/C/0.48 | HDL (-0.39) |
| 18 | *LIPG* | rs7241918 | T/G/0.17 | HDL (-1.31) |
| 18 | *MC4R* | rs12967135 | G/A/0.23 | HDL (-0.42) |
| 19 | *ANGPTL4* | rs7255436 | A/C/0.44 | HDL (-0.45) |
| 19 | *LDLR* | rs6511720 | G/T/0.10 | LDL (-6.99) |
| 19 | *LOC55908* | rs737337 | T/C/0.11 | HDL (-0.64) |
| 19 | *CILP2* | rs10401969 | T/C/0.10 | TG (-7.83)/LDL (-3.11) |
| 19 | *APOE* | rs439401 | C/T/0.36 | TG (-5.50) |
| 19 | *APOE* | rs4420638 | A/G/0.20 | LDL (7.14)/HDL (-1.06) |
| 20 | *MAFB* | rs2902940 | A/G/0.28 | LDL (-0.98) |
| 20 | *TOP1* | rs6029526 | T/A/0.48 | LDL (1.39) |
| 20 | *HNF4A* | rs1800961 | C/T/0.04 | HDL (-1.88) |
| 22 | *UBE2L3* | rs181362 | G/A/0.22 | HDL (-0.46) |
| 22 | *PLA2G6* | rs5756931 | T/C/0.36 | TG (-1.54) |

^1^The risk allele is marked. For a few of the SNPs risk allele are different depending on the trait (PP1R3B [HDL:A; LDL:G], FADS123 [TG, HDL:T; LDL:C], LIPC [HDL:G; TG:A]); however the risk allele was defined as the association with the lead trait
